# Barriers and Facilitators of the Use of Computerized Critical Care Information Systems in the Intensive Care Unit: Qualitative Interview Study

**DOI:** 10.2196/49254

**Published:** 2025-08-22

**Authors:** Alica Thissen, Eduardo Salgado-Baez, Daniel Fürstenau, Renate Delucchi Danhier, Christian Meske, Pauline Kuss, Stefan Angermair, Claudia Spies, Felix Balzer

**Affiliations:** 1 Institute of Medical Informatics Charité – Universitätsmedizin Berlin Berlin Germany; 2 Department of Anesthesiology and Intensive Care Medicine Charité – Universitätsmedizin Berlin Berlin Germany; 3 Core Unit Digital Medicine & Interoperability Berlin Institute of Health at Charité Charité - Universitätsmedizin Berlin Berlin Germany; 4 School of Business & Economics Freie Universität Berlin Berlin Germany; 5 Institute for Diversity Studies Department of Cultural Studies TU Dortmund University Dortmund Germany; 6 Institute of Work Science Ruhr-Universität Bochum Bochum Germany

**Keywords:** computerized critical care information system, intensive care unit, ICU, usability, user-centered design, co-determination

## Abstract

**Background:**

Computerized critical care information systems (CCIS) can have a range of positive to negative impacts on clinical care in ICUs and the job satisfaction of ICU staff. Key factors influencing these effects include the usability of the IT system and the level of training provided. Resistance to using the system may arise from users due to increased control imposed by the system and from insufficient participation in its development and configuration. The usability of CCIS, along with other important barriers such as co-determination, has not been thoroughly examined.

**Objective:**

The study investigates barriers to using CCIS and aims to provide actionable recommendations for manufacturing companies and hospitals to ensure the successful implementation and use of CCIS. It focuses on known usability factors while incorporating open feedback from current users to uncover new insights and identify additional usability factors not previously documented in the literature.

**Methods:**

We conducted 10 semistructured qualitative interviews with ICU staff (4 nurses and 6 doctors) from 3 ICUs at a large German university hospital. Each interview lasted 1 hour and focused on providing a collective assessment of the CCIS. The interviews were recorded, transcribed, and coded using MAXQDA and then analyzed through both deductive and inductive content structure methods.

**Results:**

A total of 86 distinct usability issues were identified and categorized into 7 main groups and 23 subgroups. The most common usability issues were (1) unclear information presentation, especially for medication; (2) overly lengthy or small interaction steps in documentation; (3) missing or scattered information across sections; (4) redundant data entry requirements; and (5) slow system speed. Additionally, other challenges associated with system usage included training, co-determination, the perception of feeling constrained, adherence to standard operating procedures, and changes in processes. Participants in the study highlighted that the level of contentment with the system has a direct influence on their job satisfaction.

**Conclusions:**

CCIS usability greatly influences satisfaction with the information technology system. Enhancing CCIS usability requires ongoing user testing and transparent employee involvement. Adequate training and standard operating procedure implementation are crucial. Investing time and financial resources in organizational frameworks and feedback mechanisms is an imperative for the successful implementation of CCIS.

## Introduction

### Background

Intensive care units (ICUs) are increasingly adopting computerized critical care information systems (CCISs) to manage the growing complexity of data in intensive care settings [1-3]. CCISs offer numerous benefits, such as enhancing the quality of care [4-6], streamlining treatment through comprehensive vital sign visualization [2,3], saving time on documentation [7-11], reducing medication errors [12-16], and adverse drug events [17,18]. Designed as sophisticated digital platforms, they interact, collect, integrate, store, and analyze extensive patient data from multiple sources, including medical devices (eg, ventilators, medication perfusers, and dialysis machines), electronic health records (EHRs; eg, demographic data and previous medical history), and laboratory systems. They can also improve staff perception of documentation quality [9] and care [4-6], leading to increased job satisfaction [19,20]. Additionally, CCISs have the potential to facilitate quality management when fully integrated into the hospital’s IT ecosystem [21]. Nowadays, these systems play a crucial role in offering a thorough, visual representation of real-time patient data for the purpose of monitoring and managing patient information. However, studies have shown mixed results regarding the impact of CCIS implementation, with some indicating no significant effects [22-26] or even negative outcomes like increased documentation time [27,28] or medication errors [29].

The International Organization for Standardization (ISO) defines software usability as the efficiency, effectiveness, and user satisfaction with which certain specific goals are achieved [30]. Software usability is a critical factor determining whether the system yields positive or negative outcomes [11,31-33]. Usability ratings are also influenced by the length of training provided [34]. Disparities in the quality of specific functions among different CCIS underscore the importance of user involvement in further development. Additionally, different professional groups may face unique challenges with CCIS [35,36], highlighting the need for more research in this area. Few studies have specifically addressed CCIS usability in ICUs [34]. Apart from the system's usability, various factors can influence its acceptance. The implementation of a CCIS may trigger resistance among staff [37], which could stem from issues related to usability. However, beyond this aspect, CCISs have the potential to elicit negative emotions such as a feeling of loss of control [38] or being controlled [39]. This can lead to “technostress” and result in decreased job satisfaction [40-44], thereby hindering effective usage and detracting from optimal performance at work. Numerous studies emphasize the significance of individual agency (the ability or state of individuals to exert power and feel in control of their lives [45]) in clinical settings and its impact on job satisfaction [46-48]. They also stress the importance of employee involvement and participation during the implementation phase [49,50] to enhance individual agency. A thorough and nuanced examination of specific systems and their impacts in particular contexts is recommended [49,51].

### Aim of the Study

This study investigates usability barriers and factors hindering effective CCIS use in ICUs, focusing on obstacles to system usage, users’ perceptions of documentation, and limitations on autonomous work. By identifying these issues, this work aims to provide actionable recommendations for software manufacturers and hospitals for the successful implementation and use of CCIS.

## Methods

The qualitative, semistructured narrative interviews were conducted between May and September 2022 at a large university hospital in Germany [52,53]. The CCIS used in the hospital was COPRA 6 from COPRA System GmbH [54]. Various studies have assessed COPRA as having moderate usability [34,36]. Initially, 4 exploratory interviews were conducted with a diverse group including a nurse, a physician, a member of the COPRA governance team, and a member of the in-house COPRA IT team. The aim of these 4 initial interviews was to gain a better understanding of the system, refine relevant guiding questions, and validate their significance based on the literature review, which helped inform the subsequent classification of study findings. Then, 10 interviews lasting approximately an hour each were conducted. Only physicians and nurses, as the primary users of the system, were invited to participate in the interviews. The aim was to conduct between 10 and 15 interviews. The decision to conclude after 10 interviews was made upon reaching content saturation, following the approach outlined by Corbin and Strauss [55]. Content saturation is defined as the recurrence of similar themes and issues, indicating that continuing recruitment will not add significant new insights.

Recruitment was conducted using flyers and through physician colleagues of members of the research team, who initiated contact verbally or via mail. Participation was voluntary, and no incentives were offered. Participants were recruited from 3 different surgical-focused ICUs. The semistructured interviews were based on a previously developed interview guide that addressed known barriers to use (such as usability, training, co-determination, agency, control, process changes, and job satisfaction; refer to Multimedia Appendix 1). Approval for this study was granted by the ethics committee of Charité—Universitätsmedizin Berlin, Germany (EA1/043/22), as well as the staff council. Prior to the study, all participants provided written informed consent. The interviews were conducted in a quiet room with a computer running COPRA by a psychologist from the research team who had experience in qualitative interviews and usability tests. Interviews were scheduled based on participant availability. While the order of questions was generally fixed, it was adjusted dynamically based on interviewee responses. In addition to the predetermined questions, participants were encouraged to discuss any relevant topics not explicitly covered. To illustrate usability issues, the system was demonstrated on the computer during the interviews. The 10 interviews were audio-recorded, transcribed, and analyzed using MAXQDA (MAXQDA by Verbi) at the end of 2022, using thematic content analysis following Kuckarzt and Rädicker’s [56] approach.

The interview guide provided a specific framework for the analysis, initially following a deductive approach where the questions served as main categories, unless new content required the addition of inductive main categories (cf. also [57]). Subcategories within the main categories were also formed inductively. For the usability aspect, subcategories were derived from pre-existing categories based on the work of von Dincklage et al [58], who developed the first validated instrument to measure CCIS usability. New subcategories were introduced or adapted inductively as well. In total, 2 subcategories were used to enhance the depth of the usability results. A second team member reviewed the analysis of the data until agreement on the categories was reached.

## Results

### Study Population

In total, 4 nurses (3 male, 75%; median age 50.5 years, range 26-60 years; median experience with COPRA 8.5 years, range 0.5-16 years) and 6 physicians (4 male, 66%; median age 29.5 years, range 27-39 years; median experience with COPRA 2.25 years, range 0.75-5 years) participated in the study. The results were extracted exclusively from the qualitative, semistructured narrative interviews.

### Background Information From the Preliminary Interviews

The hospital has used the COPRA CCIS system for over 15 years, upgrading to the latest version, COPRA 6, in 2019. The system is continuously customized by the hospital's IT department. Feedback from ward personnel is collected by specific staff members and relayed to center deputies, forming the “COPRA Governance” team of 13 members, including physicians and nurses from various ICUs. This team reviews feedback, proposes modifications, and works with the IT department to implement changes based on initial interviews. Updates are usually communicated via email through the governance team and team leads.

### Work Situation

Patient wards are equipped with bedside computers, computers in doctor and nurse workrooms, and mobile carts used during rounds, all of which are well-received. However, noise levels remain high due to frequent and loud device alarms. Caring for critically ill patients is demanding and requires intense focus, while high workloads, time constraints, limited breaks, overtime, and shift work contribute significantly to staff stress. “There is always an abundance of tasks, there is never a moment of rest” [doctor, 30s]. “A single mistake could be catastrophic” [nurse, 40s]. Documentation is often delayed, increasing the risk of errors or incomplete entries. Limited time for regular system checks further heightens risks. Despite the challenges, many find satisfaction in their roles, driven by a passion for helping others.

### Impact on Job Satisfaction

Participants linked job satisfaction to system performance, noting that a smooth, unnoticed system enhances work, while a malfunctioning one causes frustration. The barriers outlined below influence job satisfaction, with unresolved issues leading to stress, resistance, less time for patient care, and increased error risk. Respondents also emphasized that, even with a well-functioning CCIS, overall working conditions have a more significant negative impact on job satisfaction.

### Benefits of CCIS and Perception of Documentation Activities

Most respondents rate the system as satisfactory. On a scale of 1=non-satisfactory to 10=fully satisfactory, nursing staff gave a median rating of 7.5/10 (range 4-8), while physicians rated it 7/10 (range 3-9), leaving room for improvement. All participants valued documentation and preferred the CCIS over paper-based ones. Benefits included automated data retrieval, legible records, centralized patient information, and quick situational assessments. Users praised the system’s remote accessibility, detailed documentation, transparency, and therapy template suggestions, particularly for medication management. They highlighted improved patient safety through streamlined digital workflows and data protection, as well as benefits for legal purposes, billing, and quality audits.

### Potential Barriers Related to the Use of CCIS

**Figure 1:**
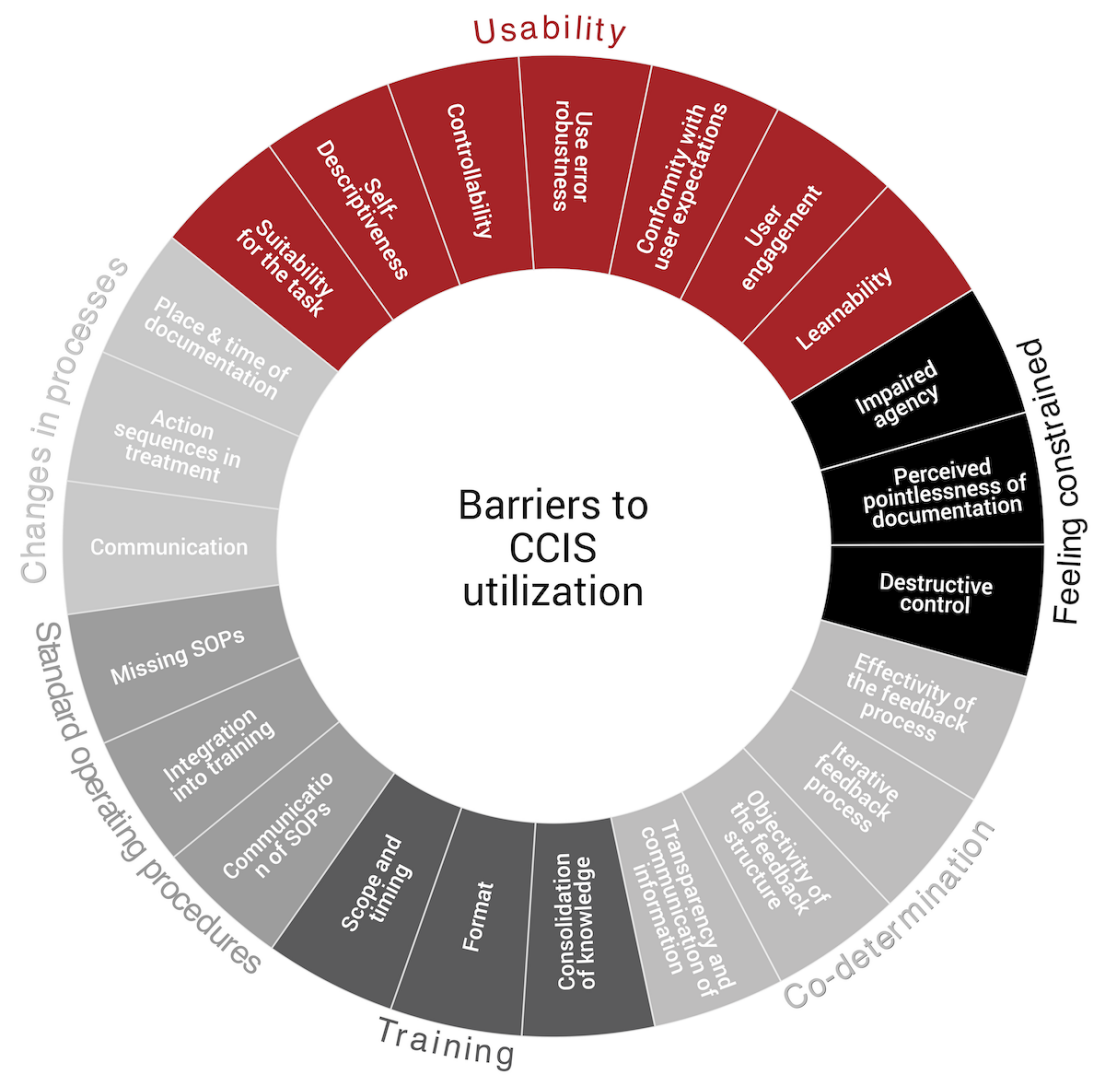
Categorization system of barriers to computerized critical care information systems (CCISs) utilization identified in the qualitative analysis. Barriers related to usability and manufacturer are shown in red, while those related to organizational units are depicted in grayscale. SOP: standard operating procedure.

After analyzing the interviews, several obstacles to effective usage were identified, highlighting key areas requiring improvement for optimal use. These obstacles were categorized into 6 primary groups, each with several subcategories. The categorization system is detailed in Figure 1 and further discussed in the subsequent sections.

### Role of Usability

The main concern addressed by the interviewees was the system’s usability, which, while generally rated satisfactory by nurses and physicians (median rating 7/10; 7.5/10), still requires substantial improvements in certain areas. The numerical satisfaction rating should not be taken as a comprehensive survey result but rather as a guide for interpreting the interview feedback. Participants expressed frustration and stress linked to a lack of user-friendliness, increased documentation time, cognitive burden, and subsequent errors. Conversely, respondents acknowledged the potential of a highly usable system to streamline tasks and save time. It is important to note that only 16 issues can be directly attributed to manufacturer specifications, particularly in medication-related areas, label and symbol clarity, and error prevention. The majority of identified problems stem from internal customization, influenced by governance decisions, or inadequate user feedback mechanisms, or a combination of manufacturer specifications and customization. This highlights the significance of well-structured internal communication channels between users and various stakeholders in achieving user-centered design.

**Figure 2:**
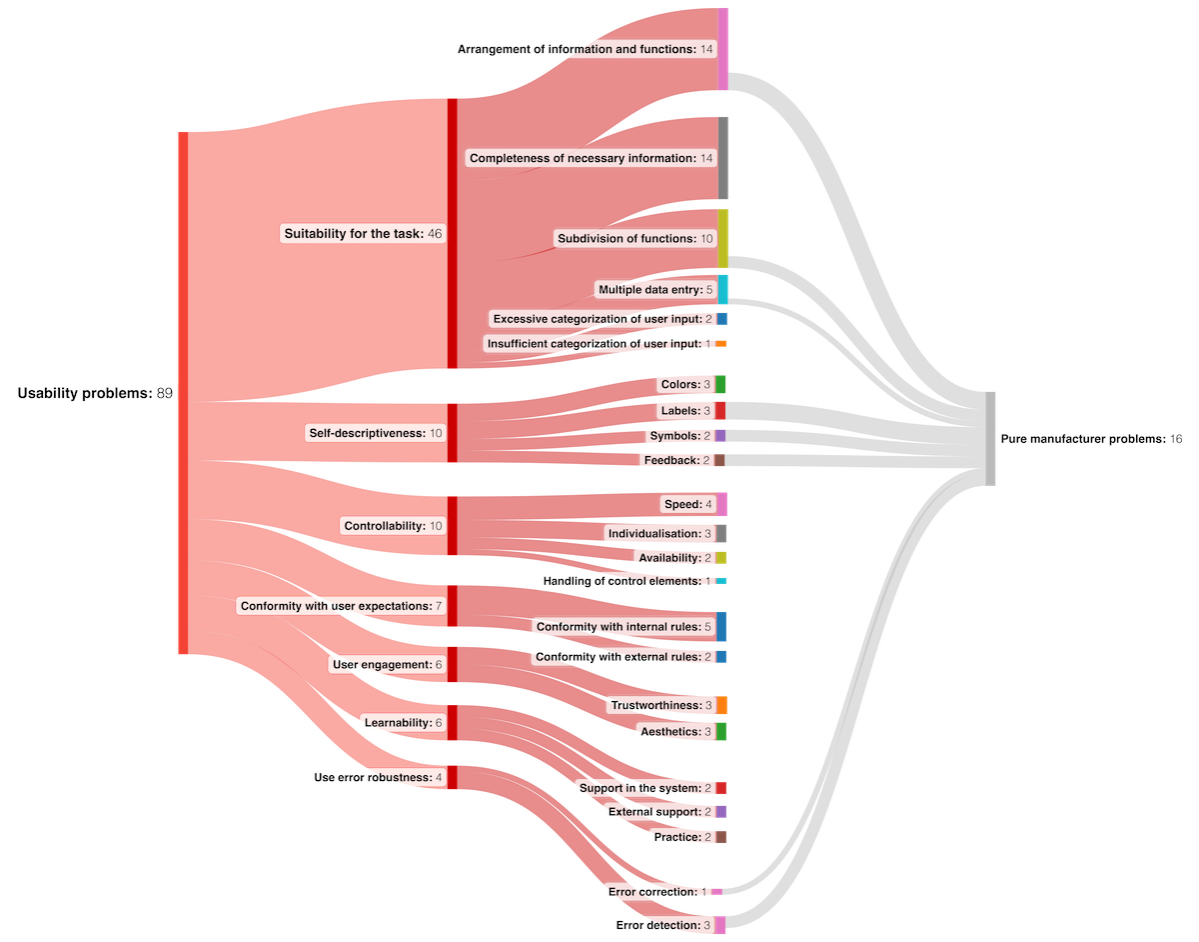
Results of the qualitative analysis of the usability problems. In total, 7 main categories and 23 subcategories of usability problems were identified.

A comprehensive list of 86 distinct usability issues was identified and organized into 23 thematic subcategories, based on the questionnaire developed by von Dincklage et al [58], with some modifications. To provide a broader overview, 7 primary categories were established, in alignment with the approach of von Dincklage et al [59] and rooted in the ISO 9241-110 dialogue principles. Figure 2 explains these categories in greater detail.

### Suitability for the Task

Most issues fell under the classification of suitability for the task. A system that is suitable for the user's tasks assists users in accomplishing their tasks by aligning operational functions and interactions with the characteristics of the task itself, rather than the technology used to execute it. A total of 46 distinct issues were identified in this category and grouped into 6 subcategories. All participants identified at least one problem within this area, resulting in a total of 77 mentions overall. Key concerns include excessive clicks, missing information, disorganized layout, repeated data entry, and inconsistent categorization, all of which hinder task efficiency and user experience.

#### Subdivisions of Functions

There was a lot of feedback about the excessive number of required clicks, particularly in the “Subdivisions of functions” category, where 8 individuals mentioned it 20 times. This category addresses the issue of needing too many clicks or steps to complete a documentation task. Criticism was focused on the need to document nursing care for different body parts in separate sections, requiring numerous clicks even for routine tasks. Users were frustrated by the system demanding additional clicks for minor deletions, such as beard care information. A participant suggested using pre-set modules that could streamline documentation processes by allowing users to check off a standard care procedure. Medication sections posed significant challenges. For example, the system did not make it possible for nursing staff to intuitively resume paused medications at a specific time with a click on the timeline. Instead, an extra click on the “now” option was required, adding to the complexity. The inability to document future medication doses also led to error messages, adding more unnecessary steps.

**Figure 3:**
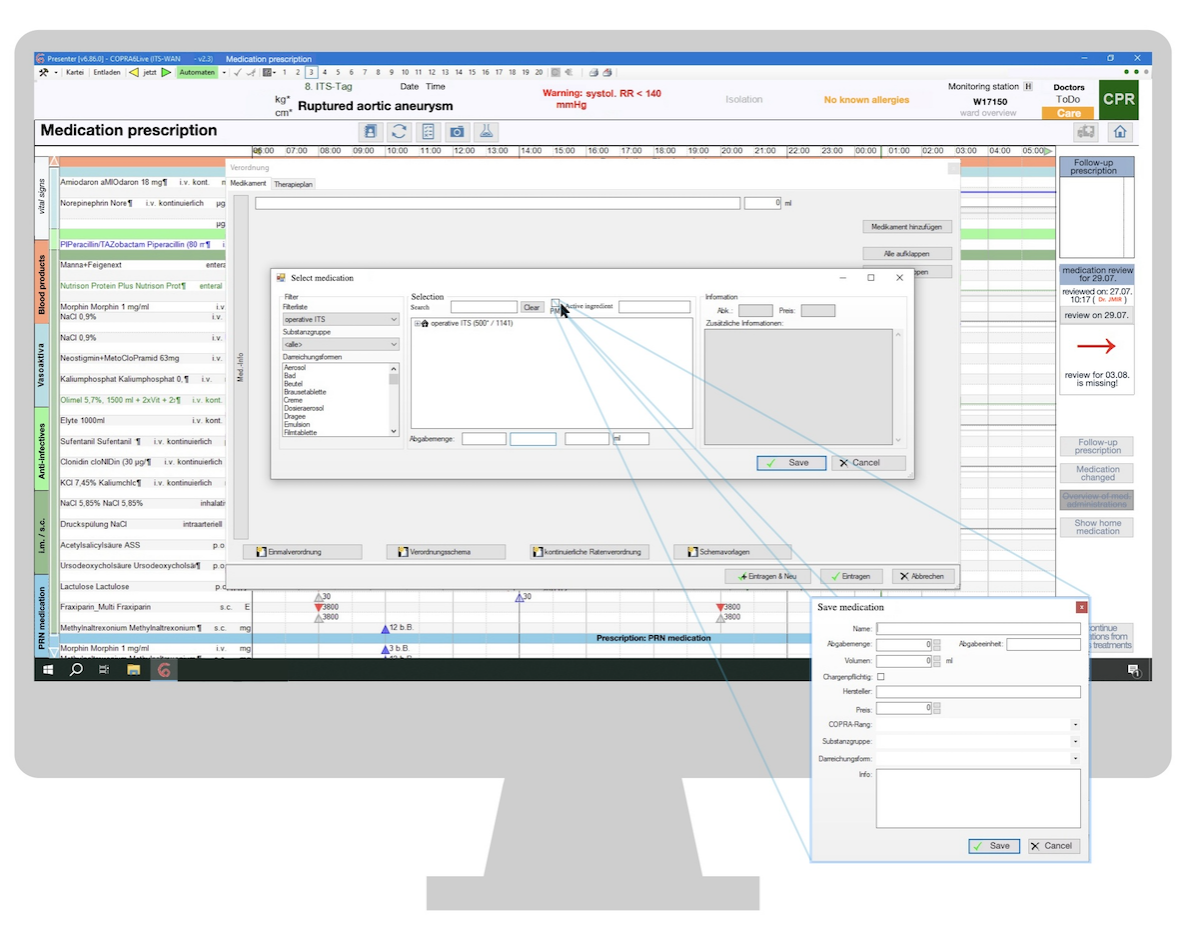
Patient-bound medication window in the medication section. ASS: Acetylsalicylic Acid; CPR: Cardiopulmonary Resuscitation; ITS: Intensive Care Unit (ICU); RR: Blood Pressure.

Nursing staff expressed concerns about not being able to access a patient's record simultaneously in both the ward and the operating room, finding repeated log-ins for continued documentation cumbersome and time-consuming. Doctors faced significant challenges when prescribing medication, particularly when required drugs were missing in the pre-selection list. In such cases, they had to use the “patient-bound medication” function, which involved manually entering detailed medication information, process many found overwhelming. This often led to gaps in information or web-based searches for details. A user interface illustrating this issue is shown in Figure 3.

Participants expressed a strong need for an integrated drug database or a link to an external source for patient-bound medication information. They also perceived the process of specifying the duration of treatment and the medication units from the preselection list as overly complex. The available unit options sometimes did not align with those used on the ward, leading to confusion between the amount of drug prescribed and its active ingredient content. This discrepancy resulted in numerous clicks, errors, and increased cognitive burden. Users suggested having the system provide medication templates or recommendations to streamline the process, which proved highly beneficial for certain medications.

#### Completeness of Necessary Information

In total, 8 users expressed frustration about missing essential information in certain sections, often due to the information being scattered across sections, unavailable within the system, or only becoming visible upon interaction (eg, clicking on an empty field or opening a drop-down menu). A doctor in their 20s suggested creating a consolidated “handover page”) to display key baseline values (eg, pulmonary, renal, and hepatic function, coagulation panel, and nutrition for doctors; blood gas analysis, glucose, potassium, and medication for nurses). There was also a desire for easier access to information not requiring a log-in, like blood gas analysis, patient positioning, and pathogens.

Other concerns included inadequate temporal display resolution, the need to search outside the CCIS for values in the hospital’s EHR, and overly small text fields that require scrolling to view complete entries. A critical issue raised by a doctor in their 30s was the system’s failure to flag new entries, forcing staff to manually check each section for updates. This could lead to overlooking important information such as new medication orders, planned interventions, laboratory values, or test results throughout the day. Suggested improvements included prompts for new entries, such as color highlighting or flashing page tabs with new information, and an overview section indicating patients with updates requiring attention.

#### Arrangement of Information and Functions

**Figure 4:**
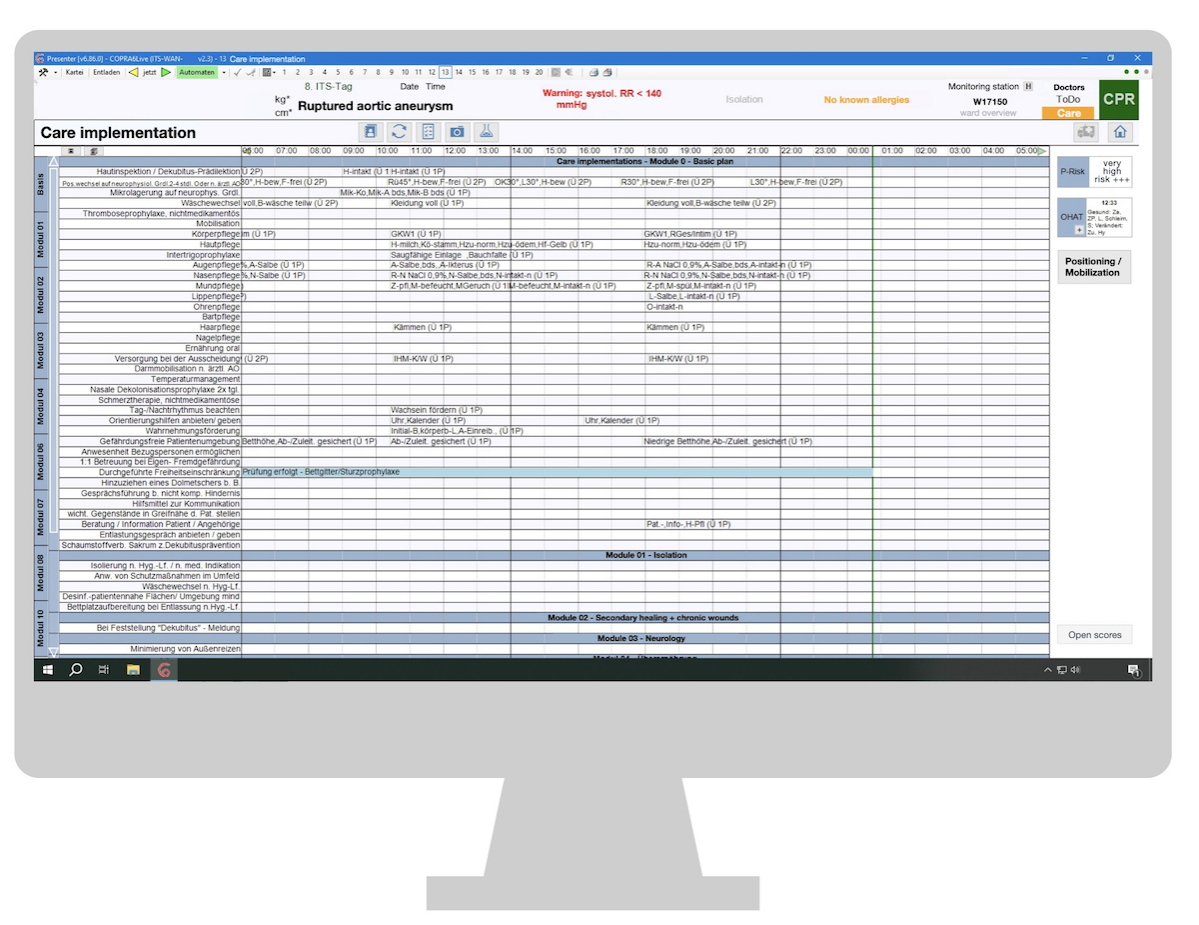
Care implementation section on the computerized critical care information system. AO: Prescription; CPR: Cardiopulmonary Resuscitation; OHAT: Oral Health Assessment Tool; RR: Blood Pressure; Ü 1P: performed by one nurse; Ü 2P: performed by two nurses.

**Figure 5:**
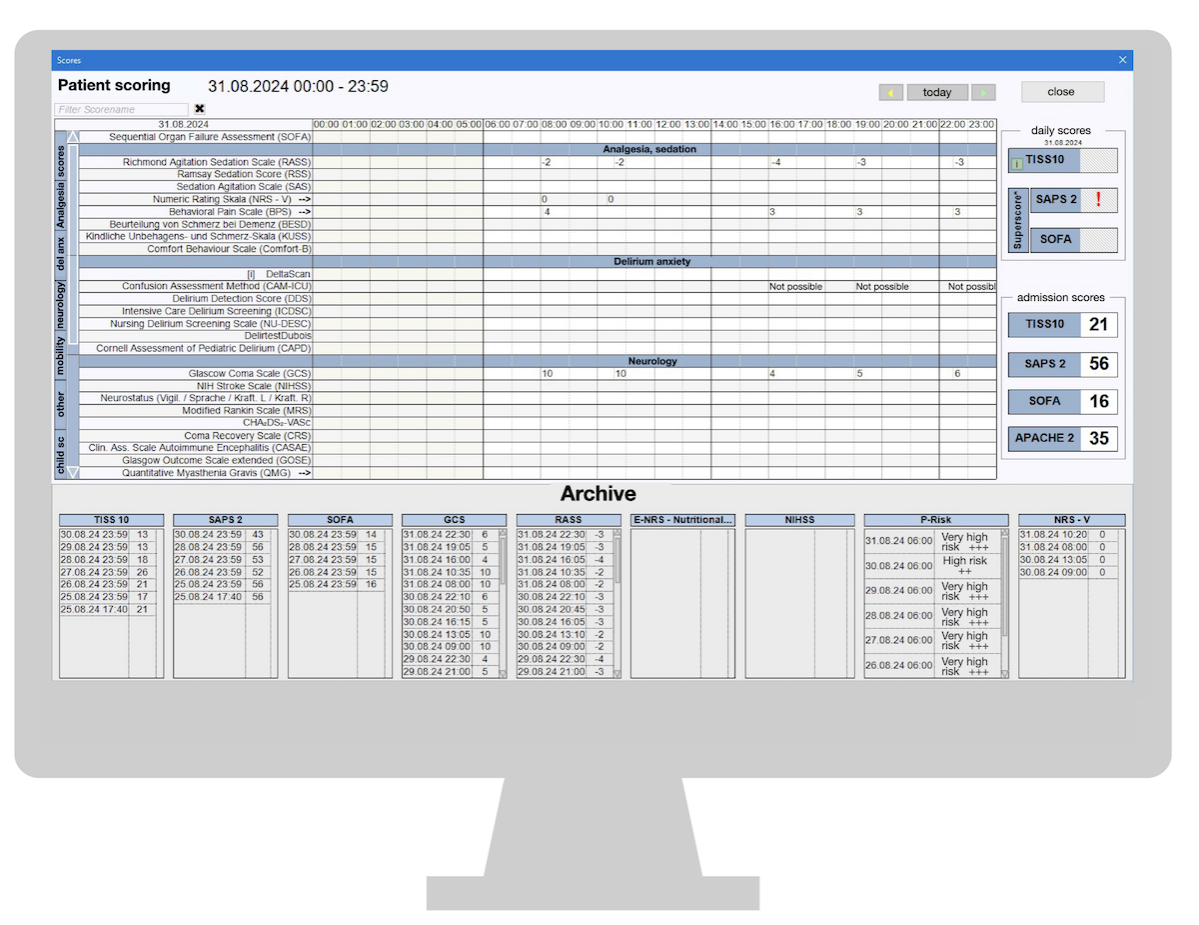
Overview of intensive care unit–relevant scores on the computerized critical care information system. SAPS 2: Simplified Acute Physiology Score II; TISS10: Therapeutic Intervention Scoring System (10-item version); APACHE 2: Acute Physiology and Chronic Health Evaluation II.

The “Arrangement of information and functions” category was also frequently mentioned, with 7 participants making mentions. Participants described the system as unclear due to the presentation of numerous information elements that are visually similar. Extensive lists and lines create challenges in maintaining visual focus, especially while performing tasks like care implementation and reviewing scores, as shown in Figures 4 and 5.

A suggested solution was enhancing the system’s customization capability, allowing for the incorporation or removal of specific values on a per-patient and user account basis. Additionally, one participant requested presenting values graphically rather than in lists, such as scores, to improve clarity and to have the system interpret and color-code values, like early warning scores, for easier recognition.

**Figure 6:**
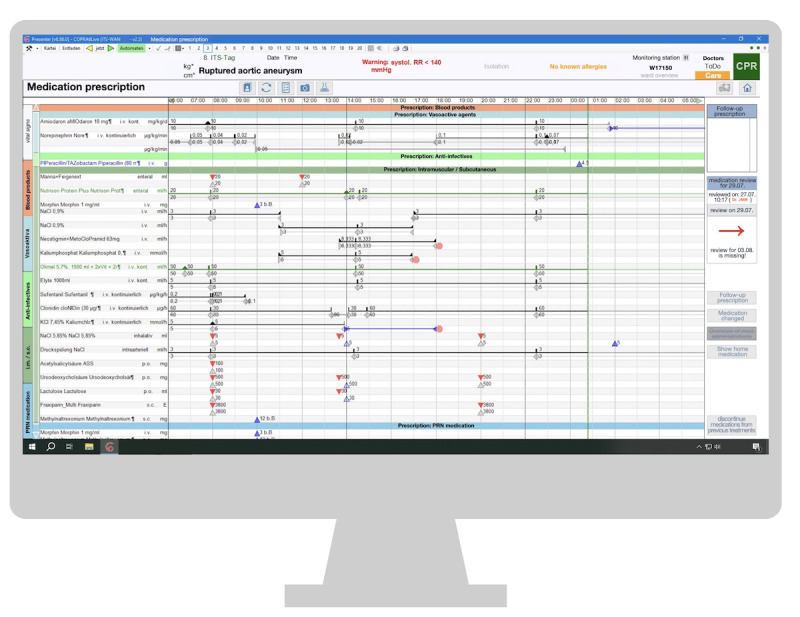
Physician's medication section on the computerized critical care information system. ASS: Acetylsalicylic Acid; CPR: Cardiopulmonary Resuscitation; PRN: Pro re nata/as-needed medication; RR: Blood Pressure.

The medication section presented significant challenges in this aspect as well. Some users found the font size to be inadequate, making it hard to recognize the information. They suggested adding a zoom feature. The close spacing of scores made it difficult to focus on a specific line. Additionally, undesired duplicated prescriptions were not displayed adjacent to the originals, leading to confusion and lengthy, disorganized lists. Users preferred a single line per drug, like the previous version of the system (COPRA 5). Distinctions between order and administration lines were subtle, due to minimal font and color differences, causing misunderstandings. The lack of alphabetical order within drug groups did not align with nurses’ usual practice of documenting prescriptions alphabetically for easier retrieval from the medication cabinet. The proximity of the medication dosage and pharmaceutical active ingredient details without clear labels caused confusion (see Figure 6).

Additional issues were noted with medication prescriptions, particularly the confusing formatting of the order list. New prescriptions appear at the bottom instead of the top, requiring scrolling and giving the impression that the system has not executed the most recent order.

#### Multiple Data Entry

In total, 5 individuals (10 mentions) expressed frustration with repeatedly entering the same information across different sections of the system. The inability to open 2 CCIS windows simultaneously was also seen as a barrier. Participants suggested automatically taking over the selection values already entered in other sections (eg, free text boxes in daily progress notes) and automatic synchronization of identical values between sections, such as the nursing care plan and the care implementation list. Beyond that, the interviewees also requested data synchronization across the EHR, CCIS, and medication infusion pumps through an interoperable interface.

#### Excessive Categorization of User Input

Additionally, 2 participants expressed criticism regarding the overly narrow categorization of information in certain instances. For example, interviewees suggested adding a textbox with free-form input for the documentation of secretions (eg, saliva, urine, and blood) instead of predefined categories.

#### Insufficient Categorization of User Input

Additionally, 2 individuals raised concerns regarding the system’s lack of adequate categorization of information in free text. They also perceived the available categories for selection as inappropriate. Specifically, issues were noted regarding the categorization of urine-related information. Criticism was expressed about the lack of consistent time stamps in the free text of the daily progress notes section. While users can add time and name stamps, it is not mandatory, leading to missing time details, especially for medication administration. An automatic timestamp feature in free text could address this issue.

### Self-Descriptiveness

Self-descriptiveness refers to the interactive system's ability to provide relevant information to the user, making its functions and usage immediately clear without unnecessary interactions.

A strong level of self-explanation in the system supports the learning process, which is especially beneficial for new users or professionals who do not regularly engage with the system, such as consultants from different specializations. In this case, 5 participants pointed out 10 distinct issues, which we categorized into 4 subgroups.

Key concerns included unclear color schemes, confusing labels and abbreviations, ambiguous symbols, and insufficient feedback, all of which reduce the system’s intuitiveness and ease of learning, especially for new and infrequent users.

#### Labels (and Abbreviations)

Unclear labeling can hinder users from becoming familiar with functions and discovering them intuitively. In total, 2 individuals criticized several labels as unintuitive (3 mentions). One example mentioned was “Automaten” (English: automatic device), which appears green on the screen when data is correctly and automatically transmitted from different devices to the CCIS. Another example was “Entladen” (English: unload), which misleads the user into thinking they closed a case while they just temporarily unloaded a patient file from the system. Participants also mentioned the abbreviation “PM,” which stands for patient-bound medication but is frequently misunderstood to mean *post meridiem*, as seen in Figure 3.

#### Symbols

In some cases, symbols are not inherently clear, like the triangles used in the medication section to signify prescriptions and medication administration. Similarly, the significance of varying line thicknesses in the medication section is not immediately apparent (see Figure 4).

#### Feedback

At times, the system feedback lacks clarity. For instance, it may not clearly indicate when devices (“Automaten”) are not sending data or when the system has completed loading data upon accessing a section or record, which is particularly concerning in long-stay cases.

#### Colors

In total, 2 individuals raised concerns 3 times about the lack of clarity in the interpretation of colors within the system. They mentioned the ambiguity of colors used for drug names or medication infusion pumps. One proposed solution was to adopt the color scheme of the *Deutsche Interdisziplinäre Vereinigung für Intensiv- und Notfallmedizin* (English: German Interdisciplinary Association for Intensive and Emergency Medicine) as a reference. Additionally, the varying colors assigned to the tasks of different occupational groups, which are shown on the display, were deemed unclear.

### Controllability

Controllability refers to the capability of the interactive system to enable the user to retain command over the user interface and interactions, encompassing the pace, sequence, and customization of the user-system engagement. A total of 10 distinct issues were mentioned by 5 respondents and categorized into 4 subgroups.

Key concerns included the need for personalization options, difficulties handling control elements, slow system speed, and limited availability of critical information when mobile or during system crashes, all of which hinder workflow efficiency and user control.

#### Individualization

One individual expressed the necessity for personalizing lists, which includes actions such as drop-down menus, removing items from lists, and saving unique settings for a single user.

#### Handling of Control Elements

In total, 2 individuals indicated that operating the system is challenging and cumbersome in certain areas. They found it hard to select a precise time by clicking on the medication sections’ timeline.

#### Speed

One major issue with the system is its slow speed, as mentioned by 4 individuals. Criticism often revolves around the extended time taken for loading or saving patient data, which hampers workflow, especially in the case of long-term ICU patients.

#### Availability

Participants stated concerns about not being able to access or input vital information while on the move within the facility. This may lead to a risk of forgetting to input important information as well as to critical issues during emergency situations. One individual also expressed a wish to use the system on mobile tablets, such as while en route to a procedure or X-ray. Another issue stressed by interviewees is the system freezing or the computer crashing, which is particularly distressing during critical situations.

### Use Error Robustness

The interactive system demonstrates resilience to user errors by helping users prevent mistakes and handling identifiable errors with tolerance while also aiding users in rectifying those errors. In total, 4 distinct issues indicated by 4 participants were identified, which were classified into 2 subgroups.

Key concerns included the cumbersome process of error correction, particularly in the case of medication prescriptions, and the need for improved error detection, such as earlier identification of discrepancies and plausibility checks for unrealistic readings or dangerous drug combinations.

#### Error Correction

At times, the system presents challenges when users attempt to rectify errors. Concerning medication prescriptions, 3 users expressed dissatisfaction with the lengthy and cumbersome process of modifying a prescription.

#### Error Detection

The system can potentially offer increased assistance in identifying errors (mentioned by 1 individual 3 times). One critique was that after numerous interactions, the system only detects a discrepancy between care planning and care validation, resulting in a delayed error message and the need for revalidation after adjusting the list. Additionally, there was a suggestion for the system to conduct plausibility checks, such as flagging illogical or impossible blood pressure readings and potentially harmful drug combinations.

### Conformity With User Expectations

The alignment of the system with user expectations refers to its ability to act predictably based on the usage context and widely accepted standards. In total, 7 distinct issues mentioned by 5 participants were identified and categorized into 2 subgroups.

Key concerns included challenges adapting to new layouts or rules in system updates, inconsistent design practices, and deviations from widely accepted digital design norms, such as unexpected checkbox behavior and non-standard information placement. These issues create confusion and increase cognitive effort for users.

#### Conformity With Internal Rules

Participants described changing old rules, page layouts, or assignments in an update or new system version as highly challenging. Employees who have been using the system for a long time become accustomed to a specific layout, making it difficult to adapt to a new routine. One nurse mentioned still searching for information on the incorrect page years after a system update. The system displays partial inconsistency in its design, with 3 individuals mentioning this issue a total of 5 times. An example of this inconsistency is the use of grayed-out areas versus white areas to indicate non-editable content, a rule that is not consistently applied.

#### Conformity With External Rules

Due to the prevalent integration of digital systems and devices in daily life, users have become accustomed to specific design principles that are occasionally violated by the system. Users find it counterintuitive that the total fluid balances are displayed at the bottom left instead of the conventional bottom right placement in tables. Additionally, certain elements like checkboxes deviate from their typical functionality. Normally, a checkbox indicates a yes or no response, but users are surprised when clicking the box results in opening a new window or immediately altering the interaction field, as seen with the “PM” checkbox for medication. These inconsistencies in design impose an extra cognitive burden on users and necessitate challenging adjustments.

### User Engagement

User engagement is facilitated when the interactive system presents functions and information in a welcoming and motivating way, encouraging ongoing and continued interaction with the system. There were a total of 6 distinct issues mentioned by 5 individuals, classified into 2 subcategories.

Key concerns included compromised trustworthiness due to system errors like inaccurate abbreviations and miscalculated scores as well as poor and outdated aesthetics, calling for a more modern, visually appealing design to improve the overall experience.

#### Trustworthiness

Trustworthiness is compromised in certain instances when users encounter inaccuracies within the system. In total, 3 individuals reported instances of errors, such as an inaccurate abbreviation for drops, missing scores in the scores section, and scores that were partially miscalculated by the system.

#### Aesthetics

The system is considered unattractive or even unsightly. The clinical examination section was referred to as “uninspired” by 2 individuals, mentioned 3 times. A more modern and visually appealing design could enhance user experience. The plain start list for selecting patient files in a ward is also seen as uninspired. Instead, a layout like the bed overview, which shows the room layout along with patients, is preferred.

### Learnability

The interactive system exhibits learnability by enabling users to discover its features and functionalities, encouraging exploration, reducing the learning curve, and helping whenever necessary. The study identified 3 key issues related to the system’s learnability that were raised by 5 participants and classified into 3 subcategories.

Key concerns included overwhelming information and inconsistent interface behavior, a lack of interactive support like prompts, insufficient promotion of the support hotline, and limited opportunities for practice with simulated data, making it difficult for users to become familiar with the system efficiently.

#### Support in the System

Users find the system overwhelming at first due to the large amount of information presented simultaneously, the system’s lack of clarity, and inconsistent interface behavior. The absence of interactive support, such as prompts or hover explanations, was highlighted as a barrier to learning. Participants believed that incorporating these features could improve usability at initial use.

#### External Support

One participant noted that the 24/7 support hotline was not clearly and sufficiently promoted.

#### Practice

In total, 2 participants emphasized the need for easier access to practice sessions with simulated data when they are not required to provide patient care. Although these sessions were available, they were not well integrated into the system.

**Figure 7:**
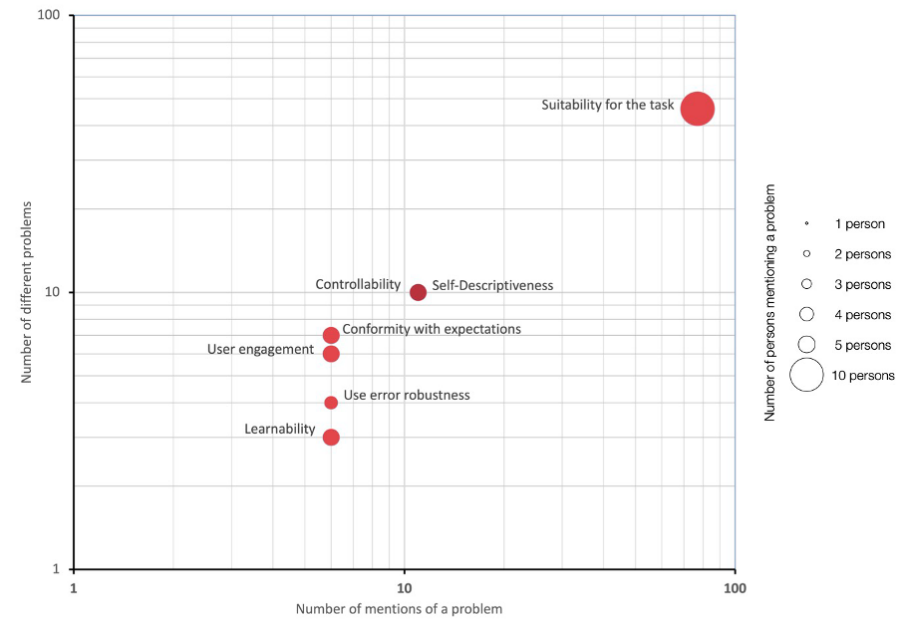
The 7 primary categories of usability problems identified in this study, using circular representations. Each circle’s size corresponds to the number of respondents who reported a problem within that category. Notably, the categories of controllability and self-descriptiveness are depicted in darker hues to indicate their overlap. These categories are positioned on a two-dimensional grid, with the x-axis denoting the frequency of mentions for any problem within a category (where multiple mentions by different individuals are counted separately, while multiple mentions by the same individual are counted once). The y-axis represents the total count of distinct usability issues within a category. Both axes employ a logarithmic scale due to the prevalence of suitability-related issues across all 3 variables.

The main categories of usability problems identified in the study are visually summarized in Figure 7, along with their respective frequencies of problems reported by the participants.

#### Additional Important Barriers

Results pertaining to barriers related to organizational units (co-determination, standard operating procedures, feeling constrained, and changes in processes) have been included in Multimedia Appendix 2

## Discussion

### Summary of the Results and Derived Recommendations

The main objective of this study was to identify barriers hindering the effective use of a CCIS in 3 ICUs of a large German university hospital. We primarily focused on usability issues and other influencing factors from the literature that could affect user satisfaction [60-63], such as co-determination, training, and personal agency. The findings aimed to identify key barriers to usage and suggest improvements for CCIS design and implementation. Additionally, the results offer insights into why a CCIS might contribute to prolonged documentation times, documentation errors, and user resistance.

In the demanding ICU environment, a CCIS is expected to streamline tasks and free up time for activities beyond documentation [64,65]. Despite potential criticisms, all participants in this research found the CCIS helpful and preferred it over traditional paper-based documentation.

The primary issue was the system's usability, which needed improvement in several areas. Concerns include unclear information presentation, cumbersome interaction steps, missing or fragmented information, redundant data entry, and slow speed. According to the participants of this study, prioritizing usability and customization is therefore crucial to improving CCIS acceptance, job satisfaction, and the delivery of patient care.

On the other hand, participants recognize the potential of a highly usable system to simplify tasks and save time. Both physicians and nurses expressed similar overall satisfaction, though their critiques reflected their different responsibilities and usage of the CCIS. Nurses prioritize a clearer medication section and less detailed nursing documentation, while physicians are concerned about medication ordering, duplicate entries, and cluttered patient parameter overviews. Both groups were troubled by the system’s speed and the lack of notifications for new entries. It is crucial for manufacturers to focus on user-centered design and conduct systematic, iterative usability tests [66,67]. Close collaboration between software developers and health care facilities is essential to incorporate valuable end-user feedback [68].

For clinics with in-house CCIS adaptations, it is crucial to establish an efficient feedback structure for iterative user testing. This makes it possible to continuously evaluate user requirements and obstacles, facilitating effective enhancements. Regular usability tests with small user cohorts are more beneficial than waiting for department feedback. Customized documentation systems must balance user needs, billing regulations, customization limits, legal considerations, EHR compatibility, and IT team requirements [69,70]. During digital transformation, clinics should avoid noninteroperable solutions to prevent future issues [71,72].

Adequate, timely training and practice are essential for smooth system use and error reduction. Training should be consistent, in small groups, and assessed with employee input. Sole reliance on self-training through web-based resources can be overwhelming. The effectiveness of communication about training and support options should be universally assessed [73,74].

Moreover, clear standard operating procedures (SOPs) and alignment with ward or clinic processes are essential for smooth system use. SOPs enhance usage and prevent errors, so they should be implemented with system deployment, integrated into training, and easily accessible [70]. They also make it possible to monitor workflow impact during implementation and adjust the system design as needed. To ensure that the system aligns with existing work processes, prioritizing a user-centric design is essential [75].

Active user participation through co-determination in adjustment processes is crucial to preventing resistance. Without it, employees may feel frustrated and constrained. This applies to both usability and SOPs. Transparently communicating processes and decision-making criteria and engaging employees in iterative system enhancements, is recommended [76]. Adequate IT support should be available to promptly implement changes. A structured, impartial feedback mechanism, potentially supported by a neutral user experience researcher, can streamline feedback collection and act as a liaison between staff and developers [77-79].

**Figure 8:**
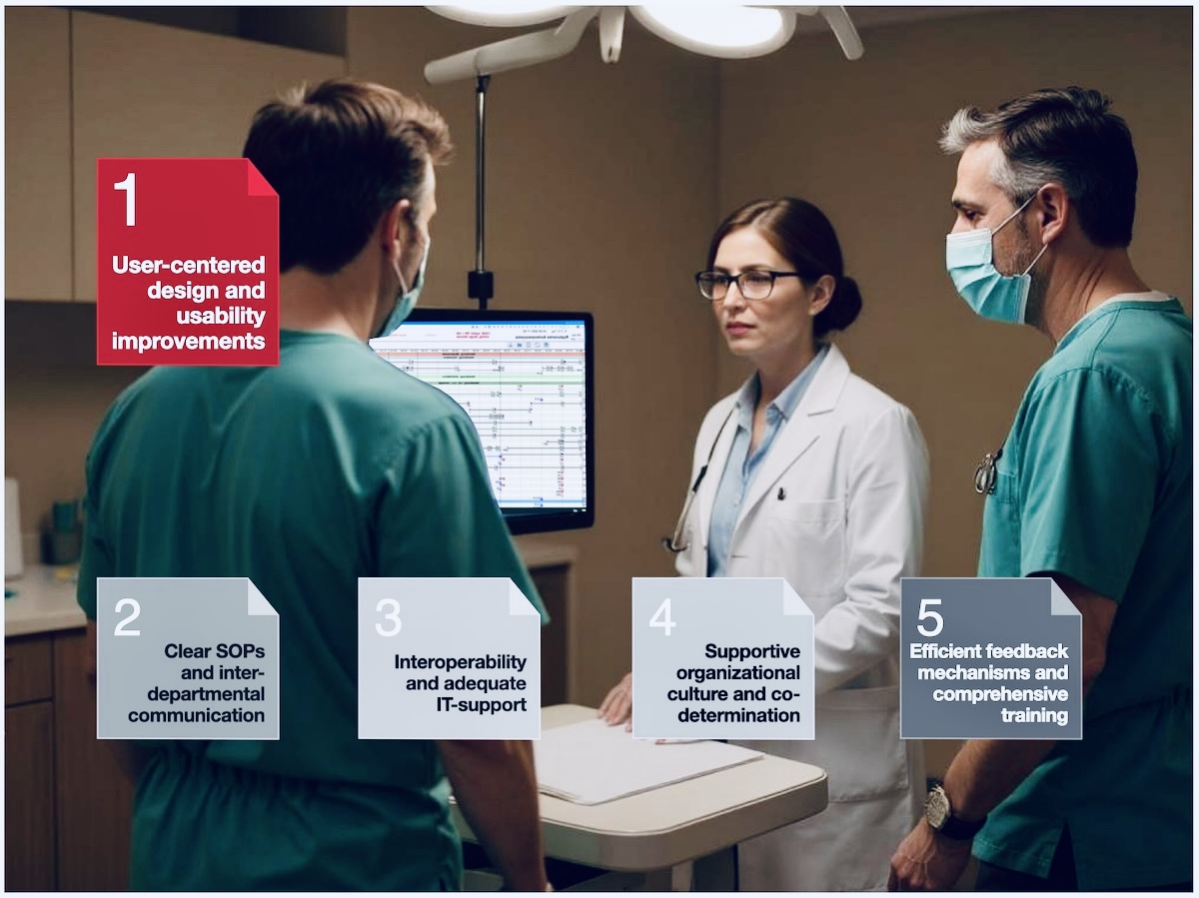
Visualization of key actionable recommendations for usability and manufacturers (in red) and organizational units (in grayscale) to ensure the successful use of computerized critical care information systems. SOPs: standard operating procedures. Artificial Intelligence-generated image, in response to the request "a realistic hospital scene inside a modern examination room. Three medical professionals are gathered around a small rolling workstation with a monitor. Two of them, male doctors in green surgical scrubs and masks, stand on either side of a female doctor in a white coat, glasses, and a light blue shirt. Overhead surgical lights illuminate them. The background shows beige walls, cabinets, a countertop with medical supplies, and a small sink. The mood is serious and professional, with warm indoor lighting and high detail, photorealistic style" (Generator: DALL•E, OpenAI; June 28, 2025. Requestor: Renate Delucchi Danhier).

Digital documentation increases control awareness, so a supportive culture toward mistakes is vital to prevent stress or user resistance [80]. It is crucial to clearly communicate the purpose of documentation to avoid confusion and the feeling of being constrained by the system [81,82]. It is also important to evaluate the necessity and format of documentation and consider flexible billing code interpretations to reduce requirements. These key actionable recommendations for the successful use of CCIS described earlier are presented and summarized in Figure 8.

### Limitations

This study had several limitations. First, it was focused exclusively on nurses and physicians. Including other professionals, such as respiratory therapists or physiotherapists, could have offered more comprehensive insights into system usability.

Second, while usability is a crucial factor in user satisfaction, other organizational aspects can also play a significant role in system acceptance. Although the introduction addressed usability criteria, the study design did not specifically evaluate efficiency, effectiveness, or user satisfaction. The 10 semistructured interviews provided an overview of usage patterns but did not delve into detailed user interactions with the system. To carry out such an analysis, classic task-based evaluations should be conducted to differentiate between the system’s actual performance and users’ subjective opinions. Additionally, the use of purposeful sampling may have introduced bias, as participants were not randomly selected. Third, although the study identified significant usability issues in the CCIS, it did not aim to provide a comprehensive evaluation of it, including positive aspects. The urgency of these issues was inferred from the frequency of their mention, but a larger, more quantitative assessment is necessary for statistical validation. Finally, the results are specific to the system and hospital where the study was conducted, which may limit their broader applicability.

Despite these limitations, the findings remain valuable, as many of the identified issues are likely relevant in other contexts, especially as the digitalization of processes and documentation in ICUs continues to grow in importance for both medical and nursing practices. This study can serve as a foundation for addressing potential barriers and enhancing CCIS usability across diverse health care settings in the future.

### Comparison With Prior Work and Outlook

This study is the first to qualitatively address the challenges related to the usability of the CCIS COPRA 6, complementing previous research that emphasizes the need for a thorough assessment of digital system usability and the importance of examining co-determination preferences in specific clinical settings [34,49,83]. It emphasizes the interconnectedness of usability issues, training requirements, co-determination structures, process adjustments, and their influence on job satisfaction, addressing a gap in the existing literature on the use of CCIS in critical care. It offers valuable insights for decision makers and developers within the clinical field. The findings align with research on usability in other clinical systems, advocating for a human-centered design approach [84-86]. They suggest that systems, procedures, and guidelines should adhere to this principle. Additionally, the results support the call for enhanced employee training on digital systems, consistent with previous studies [87].

Future research should thoroughly evaluate COPRA's usability, especially in medication management. Additionally, exploring optimal feedback processes, measuring job satisfaction, and effective communication methods within clinics could provide valuable insights.

Given the shortage of skilled workers in clinics, addressing identified barriers is crucial for job satisfaction, a healthy work environment, and long-term employee retention. However, the successful implementation of proposed improvements is closely tied to the challenge of maintaining the economic efficiency of clinics. It is important to acknowledge that adequate resources are necessary for effective system and process adjustments during digitization.

## Appendix

Multimedia Appendix 1 Interview guide questions.

Multimedia Appendix 2 Barriers related to organizational units (co-determination, standard operating procedures, feeling constrained, and changes in processes).

## Abbreviations

CCIS: computerized critical care information system
EHR: electronic health record
ICU: intensive care unit
ISO: International Organization for Standardization
SOP: standard operating procedure

## References

[1] Levy MM (2004). Computers in the intensive care unit. J Crit Care.

[2] Martich GD, Waldmann CS, Imhoff M (2004). Clinical informatics in critical care. J Intensive Care Med.

[3] Varon J, Marik PE (2002). Clinical information systems and the electronic medical record in the intensive care unit. Curr Opin Crit Care.

[4] Fraenkel DJ, Cowie M, Daley P (2003). Quality benefits of an intensive care clinical information system. Crit Care Med.

[5] Shekelle P, Morton S, Keeler E (2006). Costs and benefits of health information technology. Evid Rep Technol Assess (Full Rep).

[6] van Schijndel RJMS, de Groot SDW, Driessen R, Ligthart-Melis G, Girbes A, Beishuizen A, Weijs P (2009). Computer-aided support improves early and adequate delivery of nutrients in the ICU. Neth J Med.

[7] Ballermann M, Shaw N, Mayes DC, Gibney N, Ho SK, Hagtvedt R (2010). Intensive care unit nurse workflow during shift change prior to the introduction of a critical care clinical information system. J. Health Inform.

[8] Bosman R (2009). Impact of computerized information systems on workload in operating room and intensive care unit. Best Pract Res Clin Anaesthesiol.

[9] Donati A, Gabbanelli V, Pantanetti S, Carletti P, Principi T, Marini B, Nataloni S, Sambo G, Pelaia P (2008). The impact of a clinical information system in an intensive care unit. J Clin Monit Comput.

[10] Wong DH, Gallegos Y, Weinger MB, Clack S, Slagle J, Anderson CT (2003). Changes in intensive care unit nurse task activity after installation of a third-generation intensive care unit information system. Crit Care Med.

[11] Mador RL, Shaw NT (2009). The impact of a Critical Care Information System (CCIS) on time spent charting and in direct patient care by staff in the ICU: a review of the literature. Int J Med Inform.

[12] Bates DW, Teich JM, Lee J, Seger D, Kuperman G, Ma'Luf N, Boyle D, Leape L (1999). The impact of computerized physician order entry on medication error prevention. J Am Med Inform Assoc.

[13] Cordero L, Kuehn L, Kumar RR, Mekhjian H (2004). Impact of computerized physician order entry on clinical practice in a newborn intensive care unit. J Perinatol.

[14] Kaushal R, Shojania KG, Bates DW (2003). Effects of computerized physician order entry and clinical decision support systems on medication safety: A systematic review. Arch Intern Med.

[15] van Rosse F, Maat B, Rademaker C, van Vught AJ, Egberts A, Bollen C (2009). The effect of computerized physician order entry on medication prescription errors and clinical outcome in pediatric and intensive care: a systematic review. Pediatrics.

[16] Warrick C, Naik H, Avis S, Fletcher P, Franklin B, Inwald D (2011). A clinical information system reduces medication errors in paediatric intensive care. Intensive Care Med.

[17] Salgado-Baez Eduardo, Heidepriem Raphael, Delucchi Danhier Renate, Rinaldi Eugenia, Ravi Vishnu, Poncette Akira-Sebastian, Dahlhaus Iris, Fürstenau Daniel, Balzer Felix, Thun Sylvia, Sass Julian (2025). Toward interoperable digital medication records on Fast Healthcare Interoperability Resources: development and technical validation of a minimal core dataset. JMIR Med Inform.

[18] Leung AA, Keohane C, Amato M, Simon SR, Coffey M, Kaufman N, Cadet B, Schiff G, Zimlichman E, Seger DL, Yoon C, Song P, Bates DW (2012). Impact of vendor computerized physician order entry in community hospitals. J Gen Intern Med.

[19] Murff H, Kannry J (2001). Physician satisfaction with two order entry systems. J Am Med Inform Assoc.

[20] Schuld J, Schäfer T, Nickel S, Jacob P, Schilling MK, Richter S (2011). Impact of IT-supported clinical pathways on medical staff satisfaction. A prospective longitudinal cohort study. Int J Med Inform.

[21] Kumpf O (2021). Qualitätsindikatoren in der Intensivmedizin: Hintergrund und praktischer Nutzen. Med Klin Intensivmed Notfmed.

[22] Apkon M, Singhaviranon P (2001). Impact of an electronic information system on physician workflow and data collection in the intensive care unit. Intensive Care Med.

[23] Kilgore ML, Flint D, Pearce R (1998). The varying impact of two clinical information systems in a cardiovascular intensive care unit. J Cardiovasc Manag.

[24] Marasovic C, Kenney C, Elliott D, Sindhusake D (1997). A comparison of nursing activities associated with manual and automated documentation in an Australian intensive care unit. Comput Nurs.

[25] Menke JA, Broner CW, Campbell DY, McKissick MY, Edwards-Beckett JA (2001). Computerized clinical documentation system in the pediatric intensive care unit. BMC Med Inform Decis Mak.

[26] Georgiou A, Prgomet M, Paoloni R, Creswick N, Hordern A, Walter S, Westbrook J (2013). The effect of computerized provider order entry systems on clinical care and work processes in emergency departments: a systematic review of the quantitative literature. Ann Emerg Med.

[27] Carayon P, Wetterneck TB, Alyousef B, Brown RL, Cartmill RS, McGuire K, Hoonakker PL, Slagle J, Van Roy KS, Walker JM, Weinger MB, Xie A, Wood KE (2015). Impact of electronic health record technology on the work and workflow of physicians in the intensive care unit. Int J Med Inform.

[28] Saarinen K, Aho M (2005). Does the implementation of a clinical information system decrease the time intensive care nurses spend on documentation of care?. Acta Anaesthesiol Scand.

[29] Koppel R, Metlay JP, Cohen A, Abaluck B, Localio AR, Kimmel SE, Strom BL (2005). Role of computerized physician order entry systems in facilitating medication errors. J Am Med Assoc.

[30] ISO 9241-11:2018(en), Ergonomics of human-system interaction ? Part 11: usability: definitions and concepts.

[31] Khairat S, Coleman C, Ottmar P, Bice T, Koppel R, Carson S (2019). Physicians' gender and their use of electronic health records: findings from a mixed-methods usability study. J Am Med Inform Assoc.

[32] Howe JL, Adams KT, Hettinger AZ, Ratwani RM (2018). Electronic health record usability issues and potential contribution to patient harm. J Am Med Assoc.

[33] Hudson D, Kushniruk A, Borycki E, Zuege DJ (2018). Physician satisfaction with a critical care clinical information system using a multimethod evaluation of usability. Int J Med Inform.

[34] von Dincklage F, Suchodolski K, Lichtner G, Friesdorf W, Podtschaske B, Ragaller M (2019). Investigation of the usability of computerized critical care information systems in Germany. J Intensive Care Med.

[35] Ghahramani N, Lendel I, Haque R, Sawruk K (2009). User satisfaction with computerized order entry system and its effect on workplace level of stress. J Med Syst.

[36] Suchodolski K, von Dincklage F, Lichtner G, Friesdorf W, Podtschaske B, Ragaller M (2019). Vergleich aktueller patientendatenmanagementsysteme in der intensivmedizin aus sicht der klinischen nutzer: Zusammenfassung der Ergebnisse einer deutschlandweiten Umfrage. Anaesthesist.

[37] Bhattacherjee A, Hikmet N (2017). Physicians' resistance toward healthcare information technology: a theoretical model and empirical test. Eur J Inf Syst.

[38] Lapointe L, Rivard S (2005). A multilevel model of resistance to information technology implementation. MIS Q.

[39] Manzei-Gorsky A (2007). Between representation, reorganization and control - the informational technification of intensive care units and the consequences. Int J Technol Knowl Soc.

[40] Abuatiq A (2015). Concept analysis of technostress in nursing. Int J Nurs Clin Pract.

[41] Califf CB, Sarker S, Sarker S (2020). The bright and dark sides of technostress: a mixed-methods study involving healthcare IT. MIS Q.

[42] Fumis RRL, Costa ELV, Martins PS, Pizzo V, Souza IA, de Paula Pinto Schettino G (2014). Is the ICU staff satisfied with the computerized physician order entry? A cross-sectional survey study. Rev Bras Ter Intensiva.

[43] Golz C, Peter KA, Müller TJ, Mutschler J, Zwakhalen SMG, Hahn S (2021). Technostress and digital competence among health professionals in swiss psychiatric hospitals: cross-sectional study. JMIR Ment Health.

[44] Golz C, Peter KA, Zwakhalen SM, Hahn S (2021). Technostress among health professionals - a multilevel model and group comparisons between settings and professions. Inform Health Soc Care.

[45] Napper DP, Rao A (2019). The Power of Agency: The 7 Principles to Conquer Obstacles, Make Effective Decisions, and Create a Life on Your Own Terms.

[46] De Simone S, Planta A, Cicotto G (2018). The role of job satisfaction, work engagement, self-efficacy and agentic capacities on nurses' turnover intention and patient satisfaction. Appl Nurs Res.

[47] Gottlieb LN, Gottlieb B, Bitzas V (2021). Creating empowering conditions for nurses with workplace autonomy and agency: How healthcare leaders could be guided by strengths-based nursing and healthcare leadership (SBNH-L). J Healthc Leadersh.

[48] Lunden A, Teräs M, Kvist T, Häggman-Laitila A (2019). Transformative agency and tensions in knowledge management—a qualitative interview study for nurse leaders. J Clin Nurs.

[49] Bräutigam C, Enste P, Evans M, Hilbert J, Merkel S, Öz F Digitalisierung im Krankenhaus Mehr Technik - bessere Arbeit?.

[50] Sockolow PS, Liao C, Chittams JL, Bowles KH (2012). Evaluating the impact of electronic health records on nurse clinical process at two community health sites. NI 2012 (2012).

[51] Meyer S-C, Tisch A, Hünefeld L (2019). Arbeitsintensivierung und Handlungsspielraum in digitalisierten Arbeitswelten ? Herausforderung für das Wohlbefinden von Beschäftigten?. IndBez.

[52] Adams WC (2015). Conducting Semi-Structured Interviews. Handbook of Practical Program Evaluation, 4th ed.

[53] Adeoye‐Olatunde OA, Olenik NL (2021). Research and scholarly methods: semi‐structured interviews. J Am Coll Clin Pharm.

[54] COPRA GmbH.

[55] Corbin J, Strauss A (2015). Basics of Qualitative Research. 4th ed.

[56] Kuckartz U, Rädiker S (2022). Qualitative inhaltsanalyse. Methoden, Praxis, Computerunterstützung. 5th ed.

[57] Grodal S, Anteby M, Holm AL (2021). Achieving rigor in qualitative analysis: the role of active categorization in theory buildinga. Acad Manage Rev.

[58] von Dincklage F, Lichtner G, Suchodolski K, Ragaller M, Friesdorf W, Podtschaske B (2017). Design and validation of a questionnaire to evaluate the usability of computerized critical care information systems. J Clin Monit Comput.

[59] DIN EN ISO 9241-110:2020-10, Ergonomie der Mensch-System-Interaktion_- Teil_110: Interaktionsprinzipien (ISO_9241-110:2020); Deutsche Fassung EN_ISO_9241-110:2020. Beuth Verlag GmbH.

[60] Al‐Maskari A, Sanderson M (2010). A review of factors influencing user satisfaction in information retrieval. J Am Soc Inf Sci.

[61] Hicks JP, Allsop MJ, Akaba GO, Yalma RM, Dirisu O, Okusanya B, Tukur J, Okunade K, Akeju D, Ajepe A, Okuzu O, Mirzoev T, Ebenso B (2021). Acceptability and potential effectiveness of eHealth tools for training primary health workers from nigeria at scale: mixed methods, uncontrolled before-and-after study. JMIR Mhealth Uhealth.

[62] Khairat S, Burke G, Archambault H, Schwartz T, Larson J, Ratwani RM (2018). Perceived burden of EHRs on physicians at different stages of their career. Appl Clin Inform.

[63] Zahidi Z, Peng Y, Charles P (2014). User satisfaction determinants for digital culture heritage online collections. Int J Adv Comput Sci Appl.

[64] Ballermann MA, Shaw NT, Mayes DC, Gibney RTN, Westbrook JI (2011). Validation of the Work Observation Method By Activity Timing (WOMBAT) method of conducting time-motion observations in critical care settings: an observational study. BMC Med Inform Decis Mak.

[65] Stavem K, Hoel H, Skjaker SA, Haagensen R (2017). Charlson comorbidity index derived from chart review or administrative data: agreement and prediction of mortality in intensive care patients. Clin Epidemiol.

[66] Schaaf J, Sedlmayr M, Schaefer J, Storf H (2020). Diagnosis of rare diseases: a scoping review of clinical decision support systems. Orphanet J Rare Dis.

[67] Aakre C, Kitson JE, Li M, Herasevich V (2017). Iterative user interface design for automated sequential organ failure assessment score calculator in sepsis detection. JMIR Hum Factors.

[68] Carayon P, Hoonakker P (2019). Human factors and usability for health information technology: old and new challenges. Yearb Med Inform.

[69] Walji M, Kalenderian E, Piotrowski M, Tran D, Kookal KK, Tokede O, White JM, Vaderhobli R, Ramoni R, Stark PC, Kimmes NS, Lagerweij M, Patel VL (2014). Are three methods better than one? A comparative assessment of usability evaluation methods in an EHR. Int J Med Inform.

[70] Pruitt Z, Howe JL, Krevat SA, Khairat S, Ratwani RM (2022). Development and pilot evaluation of an electronic health record usability and safety self-assessment tool. JAMIA Open.

[71] Lehne M, Sass J, Essenwanger A, Schepers J, Thun S (2019). Why digital medicine depends on interoperability. NPJ Digit Med.

[72] Iyamu I, Xu AXT, Gómez-Ramírez O, Ablona A, Chang H, Mckee G, Gilbert M (2021). Defining digital public health and the role of digitization, digitalization, and digital transformation: scoping review. JMIR Public Health Surveill.

[73] Mestre A, Muster M, El Adib AR, Ösp Egilsdottir H, Byermoen KR, Padilha M, Aguilar T, Tabagari N, Betts L, Sales L, Garcia P, Ling L, Café H, Binnie A, Marreiros A (2022). The impact of small-group virtual patient simulator training on perceptions of individual learning process and curricular integration: a multicentre cohort study of nursing and medical students. BMC Med Educ.

[74] Sega A, Bossan A, Abrams M, Cendan V, Gartland A, Nguyen D, Simms-Cendan J (2021). Improving student EHR accuracy: an analysis of training methods to better prepare students to volunteer at student-run clinics. J Stud Run Clin.

[75] Middleton B, Bloomrosen M, Dente MA, Hashmat B, Koppel R, Overhage JM, Payne TH, Rosenbloom ST, Weaver C, Zhang J, American Medical Informatics Association (2013). Enhancing patient safety and quality of care by improving the usability of electronic health record systems: recommendations from AMIA. J Am Med Inform Assoc.

[76] Pagano D, Bruegge B (2013). User involvement in software evolution practice: a case study. https://doi.org/10.1109/icse.2013.6606645.

[77] Almaliki M, Ncube C, Ali R (2014). The design of adaptive acquisition of users feedback: An empirical study.

[78] Nilsen P, Ståhl C, Roback K, Cairney P (2013). Never the twain shall meet? A comparison of implementation science and policy implementation research. Implement Sci.

[79] Zhang Z, Miehle J, Matsuda Y, Fujimoto M, Arakawa Y, Yasumoto K, Minker W (2021). Exploring the impacts of elaborateness and indirectness in a behavior change support system. IEEE Access.

[80] Waugh J (2014). Education in medical billing benefits both neurology trainees and academic departments. Neurology.

[81] Donaldson DR, Conway P (2015). User conceptions of trustworthiness for digital archival documents. Assoc Info Sci Technol.

[82] Burks K, Shields J, Evans J, Plumley J, Gerlach J, Flesher S (2022). A systematic review of outpatient billing practices. SAGE Open Med.

[83] Bitkina OV, Kim HK, Park J (2020). Usability and user experience of medical devices: an overview of the current state, analysis methodologies, and future challenges. Int J Ind Ergon.

[84] Poncette A, Mosch LK, Stablo L, Spies C, Schieler M, Weber-Carstens S, Feufel MA, Balzer F (2022). A remote patient-monitoring system for intensive care medicine: mixed methods human-centered design and usability evaluation. JMIR Hum Factors.

[85] Melles M, Albayrak A, Goossens R (2021). Innovating health care: key characteristics of human-centered design. Int J Qual Health Care.

[86] Searl MM, Borgi L, Chemali Z (2010). It is time to talk about people: a human-centered healthcare system. Health Res Policy Syst.

[87] Poncette A-S, Spies C, Mosch L, Schieler M, Weber-Carstens S, Krampe H, Balzer F (2019). Clinical requirements of future patient monitoring in the intensive care unit: qualitative study. JMIR Med Inform.

