# Supplementary material for: Barriers and Facilitators of the Use of Computerized Critical Care Information Systems in the Intensive Care Unit: Qualitative Interview Study
Source: J Med Internet Res. 2025 Aug 22;27:e49254. doi: 10.2196/49254 (PMC12413570; doi:10.2196/49254)
Supplement: Multimedia Appendix 1 [file jmir_v27i1e49254_app1.doc]

**MULTIMEDIA APPENDIX 1**

Interview guide questions

**USABILITY**

- What problems do you have in using the system in terms of usability? Please take a moment to reflect, you can also click through the system as you do so. Please try to select the 3-5 most pressing problems for you (more mentions or fewer mentions were allowed; here the usability problems were looked at together on the computer).
- How satisfied are you with the system on a scale of 1-10?

**CO-DETERMINATION**

- How can you provide feedback on the system? Would you like to have more say in this process?

**TRAINING**

- How did the training for the system go? What would you change about this process if you could?

**FEELING CONSTRAINED**

- Does working with the system sometimes create conflicts in your collaboration with colleagues?
- Do you feel controlled or limited in your power to act by working with the system?

**CHANGES IN PROCESSES**

- How are you informed about system updates or planned changes and what do you think about them?

**GENERAL QUESTIONS / JOB SATISFACTION**

- Does working with the system create other problems for you beyond usability that have not yet been addressed?
- How would you say the system use affects your job satisfaction?
