# Supplementary material for: Barriers and Facilitators of the Use of Computerized Critical Care Information Systems in the Intensive Care Unit: Qualitative Interview Study
Source: J Med Internet Res. 2025 Aug 22;27:e49254. doi: 10.2196/49254 (PMC12413570; doi:10.2196/49254)
Supplement: Multimedia Appendix 2 [file jmir_v27i1e49254_app2.docx]

#### Co-determination

The involvement of users in the design, development, implementation, and improvement of a CCIS represents a valuable opportunity to create more effective and user-friendly software by integrating the experiences and and insights of the users, an issue that was raised as relevant to all participants, but generally perceived as limited due to the indirect communication with the governance team.

##### Effectiveness of the feedback process

While it is acknowledged that the IT team faces resource constraints and cannot address every complaint, frustration ensues when highly relevant suggestions are either not acted upon or implemented at a very slow pace despite repeated requests.

##### Iterative feedback process

There was a suggestion for regular reassessment of implemented changes, such as conducting quantitative surveys among staff to assess the importance and effectiveness of system modifications. The dissemination of system updates through email is approved. Nevertheless, updates that are perceived as causing a decline in performance are sources of frustration.

##### Objectivity of the feedback structure

Additionally, there is a critical view on the filtered relay of information to the designated individual in charge on the ward, as it may be seen as arbitrary, indicating a lack of structured information forwarding processes. In this context, there was an expressed need for a digital suggestion box.

##### Transparency and communication of information

Lack of information about the reasons why implement a suggested improvement is not implemented frustrate users, which in general dislike decision made without any explanation. Insufficient information or transparency concerning processes that impact the system may result in negative assumptions or rumors undermining commitment and affecting the organization. For example, beliefs about management prioritizing cost-cutting over employee well-being during the system procurement. Additionally, some staff members were unaware of whom to contact for sharing suggestions, resulting in communication gaps including the availability of online training resources.

#### Relevance of training

All participants emphasized the importance of adequate training for a complex program like a CCIS, as it is initially seen as overwhelming and cannot be operated through intuition alone. While effective usability can diminish the need for extensive training, in the case of a complex system like a CCIS for ICUs, training will always be essential for optimal utilization of the system.

##### Scope and timing of training

It was emphasized that conducting training well in advance of service commencement is crucial. It was frequently observed that a brief introduction followed by on-the-job exploration of the program ("learning by doing") was not favored by all, as it resulted in stress, errors, and prolonged unfamiliarity with the system's functions, hindering optimal system utilization.

##### Format of training

Training in multiple small group sessions, where individuals could review cases and ask questions with an instructor, preferably in person and during duty release, received positive feedback. Many considered online familiarization with the system, such as through training videos, to be too demanding and viewed it as a complementary option to group training.

##### Consolidation of knowledge

There was a frequent request to practice using simulated data. It was suggested that such features should be incorporated into a system, and employees should be well informed about this option. There was a desire for regular training sessions to update knowledge, address daily questions, or acquire advanced skills through an advanced course. "I would be interested in 'COPRA for advanced users'" (female nurse, 53). Having a designated contact person for daily operational questions was also seen as beneficial. Additionally, a compact reference guide/manual with key functions printed out on the ward was suggested.

#### Standard Operating Procedures

Standard Operating Procedures (SOPs) are guidelines detailing the proper use of the system and the expected processes associated with its utilization. Although not directly queried, participants consistently raised the topic and emphasized the importance of SOPs in ensuring consistent and optimal system operation.

##### Communication about SOPs

First, it was noted that despite the presence of these Standard Operating Procedures (SOPs) in written format, they are deeply buried within the hospital's internal intranet, leading to a lack of awareness among staff regarding their location.

Lack of integration into training

Interestingly, not all individuals who were aware of the online location of the SOPs were able to take the time to read them, as it can feel burdensome amidst the daily activities in the ward. A more practical approach would be to incorporate them into training sessions.

##### Lack of SOPs

Furthermore, the absence of SOPs for all tasks leads to inconsistent practices within the ward. For instance, there is ambiguity regarding whether orders should only be entered into the system or also verbally communicated to colleagues. When not addressed uniformly, this inconsistency can result in overlooked orders.

A standard operating procedure (SOP) could detail the frequency and timing for conducting searches in the system for tasks. In the event of a reminder feature for new tasks within the system, adjustments to the SOP would be necessary.

Standard Operating Procedures (SOPs) should be tailored to suit the specific characteristics of the system and the prevailing culture within the ward. Fostering verbal communication among staff to enhance collegial relationships could be beneficial. Nevertheless, SOPs serve the crucial purpose of providing clear guidelines for reference in times of uncertainty. Key considerations include the level of detail required for documentation, the preferred format for free text entries, guidelines for using initials, accessing patient files in their rooms, and determining the most appropriate location for documentation to minimize errors.

Feeling constrained

This issue often arises when users perceive limitations, obstacles, confusion and a sense of being coerced by the system, due to excessive control from other colleagues or information that must be provided to the system which is not considered relevant by nurses and doctors, hindering their ability of working effectively and efficiently.

##### Destructive control

Respondents commonly perceive a sense of being monitored as a regular aspect of their role, which is not seen as burdensome but rather as a standard practice for ensuring patient safety. They are aware that the system enables more control than paper records, allowing authorized personnel to access it remotely. Senior staff appreciate the system for facilitating easier oversight. Interviewees recognize that their documentation can be scrutinized for treatment quality, billing accuracy, and potentially legal purposes. Due to its permanence, they view documentation in the CCIS as more significant than verbal or paper-based communication. While the system heightens the sense of being monitored, it does not necessarily result in increased stress or resistance. The organizational culture plays a crucial role, as individuals in environments with fear of reprisal may experience heightened stress or avoid documentation, whereas in supportive cultures, monitoring is seen as constructive rather than intrusive.

##### Documentation perceived as pointless

In certain instances, there is a desire for increased control, such as ensuring quality assurance for patients through constructive engagement in processes and open dialogue. Lengthy documentation tasks that are deemed unlikely to be reviewed can lead to resistance, as they are seen as futile and ultimately as a form of constraint.

##### Impaired agency

Employees are compelled to utilize the system provided by their employer, necessitating a period of adjustment. Thus, they rely on a system that offers good usability and meets their requirements. "You inevitably adjust to the system due to lack of alternatives," (doctor, 30’s). When the system functions well, this adaptation process is often subconscious. However, negative sentiments grow if the system presents challenges such as poor usability, sluggish performance, or frequent crashes, leading to a sense of restricted autonomy.

As the current use of CCIS is primarily limited to documentation with minimal intervention in the actual content, there is no sense of the system actively or intentionally restricting agency. The delicate balance of how much influence the system has on documentation practices without seeming intrusive is crucial, and it is important to continuously assess where the system may impede user autonomy. While this study did not find this to be a significant issue, the interaction with the system can indeed impact agency, based on the design of usability, co-determination processes, and SOPs.

Excessive documentation of routine tasks is viewed as a form of constraint. Similarly, the need for co-determination (involvement) in decision-making was expressed, and when this is lacking, autonomy is also limited. In the absence of a structured process for feedback and change requests, employees’ resort to submitting hazard reports to voice concerns to the hospital and safeguard patients. However, the effectiveness of the hazard report was deemed inadequate, resulting in dissatisfaction. Lack of clear explanations for decisions can also lead to frustration. In addition to system design, guidance on system usage can also be seen as a form of constraint.

#### Changes in Processes

Frequently mentioned in the interviews was the observation that the system's design influences work processes. Consequently, the introduction of a system leads to changes in processes, which should be viewed as possible obstacles or sources of errors.

##### Communication

Implementing new communication processes may require initial establishment. For example, after an update, accessing a record simultaneously in the ward and operating room became infeasible, requiring separate release of the record for patient transitions between these areas. This change was often overlooked, leading to issues and frustrated communications between departments, highlighting the potential for conflicts with process modifications. Consequently, a brief interdepartmental call has been adopted as a new procedure.

##### Action sequences in treatment

A non-communicated update can impact processes, for instance, a nurse positions the pillow in a specific way on the patient's bed to act as a cue for repositioning the patient, as a workaround for the system, due to slowness or login requirements.

##### Place and time of documentation

Documentation was conducted in various locations and times, such as at the patient's bedside, offering benefits like reducing forgetfulness and providing companionship. Alternatively, documentation could occur in a quieter separate room, often seen as advantageous. Online data access allowed for consolidation of documentation for multiple patients from memory in a separate room, which was convenient but raised the likelihood of errors.
